# Tailoring Immunotherapy Treatment of Synchronous Renal Cell Carcinoma (RCC) and Triple-Negative Breast Cancer (TNBC)

**DOI:** 10.1155/2019/6246209

**Published:** 2019-06-04

**Authors:** Iris Y. Sheng, Megan Kruse, Kathryn M. Leininger, Moshe C. Ornstein

**Affiliations:** Department of Hematology and Medical Oncology, Cleveland Clinic Taussig Cancer Institute, Cleveland, Ohio, USA

## Abstract

Synchronous tumors are defined as two tumors arising concurrently or within six months of each other. Reports of synchronous RCC and breast cancer are limited to nonmetastatic renal cell carcinoma (RCC) and hormone receptor-positive infiltrative ductal carcinoma. We present the first case of synchronous metastatic renal cell carcinoma and metastatic triple-negative breast cancer, managed with a novel combination of immunotherapy and chemotherapy.

## 1. Introduction

Synchronous tumors are defined as two tumors arising concurrently or within six months of diagnosis [1]. Reports of synchronous RCC and breast cancer are limited to nonmetastatic RCC and hormone receptor-positive infiltrative ductal carcinoma (IDC) treated with complete resection of each primary tumor followed by chemoradiation and hormone therapy [2, 3]. We present a case of tailored immunotherapy for a patient with concurrent metastatic RCC (mRCC) and triple-negative breast cancer (TNBC).

## 2. Case Report

**Figure 1:**
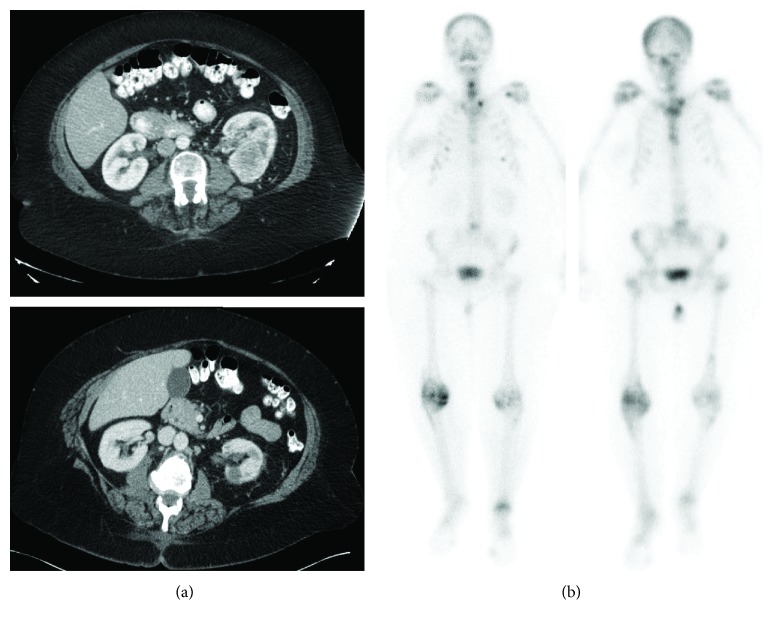
Effects of therapy on RCC ((a) pretreatment CT (10/27/2017), (b) posttreatment CT (1/17/2019)) and bony lesions ((a) pretreatment NM bone scan (10/27/2019), (b) posttreatment NM bone scan (1/16/2019)).

A 67-year-old woman presented (10/2017) with right side breast discomfort. Ultrasound demonstrated a 6.1 × 4.0 × 5.8 cm mass with no adenopathy, and biopsy showed an ER-negative, PR-negative, HER2-negative IDC. Staging computerized tomography (CT) showed bilateral pulmonary nodules (all < 3 mm), a 5 cm lower pole left kidney mass, renal vein thrombus, and innumerable sclerotic bone lesions (Figure 1). Bone marrow biopsy confirmed metastatic breast cancer. In 12/2017, she started taking capecitabine. While follow-up evaluations revealed breast softening, CT after 3 cycles of capecitabine showed no response in distant sites. She developed two new pulmonary nodules, enlarging renal mass, new retroperitoneal lymphadenopathy, and worsening osteosclerotic metastasis; capecitabine was discontinued.

For a clinical trial, breast tissue underwent genomic testing and an activating SQSTM1-RET fusion mutation was revealed. Renal biopsy (to exclude a second primary malignancy) showed clear cell RCC. The genomic analysis of the renal biopsy did not yield mutations. Ipilimumab and nivolumab was started in 5/2018. She developed treatment-related rash, which was resolved with steroids. After 4 cycles of ipilimumab/nivolumab (7/2018), CT showed partial response with a resolution of lung nodules and shrinkage of the RCC primary tumor, enlarging adenopathy and worsening bony metastasis. A clinical breast exam was normal. Given mixed response, nivolumab maintenance was implemented and nab-paclitaxel was added. CT (10/2018) showed partial response with improved adenopathy, stable renal lesion, and stable bony lesions. The patient currently remains on this combination of nivolumab and nab-paclitaxel with her last CT (Figure 1) showing stable disease (current duration of therapy: 10 months).

## 3. Discussion

When two synchronous metastatic tumors are diagnosed, genomic testing can help direct the therapy if overlapping mutations exist. In the current case, however, both primary tumors had different genomic profiles and a more nuanced approach was required.

Unlike TNBC, RCC does not respond well to chemotherapy. To date, there are no immunotherapy agents that dually treat RCC and breast cancer. However, given emerging data for immunotherapy in TNBC and strong data for the combination of ipilimumab/nivolumab in mRCC, the decision was made to treat both tumors with an immunotherapy approach [4]. The patient's initial response to therapy as highlighted above demonstrated a treatment response in the RCC lesions (kidney and lung nodules) but not in the biopsy-proven breast cancer lesions in the bone. When the patient's disease progressed, results from the Impassion130 trial were released, which showed superior ORR (56% vs. 46%) and decreased disease progression/death by 20% when nab-paclitaxel was added to atezolizumab compared to standard chemotherapy in the treatment of TNBC [5]. Thus, we continued the maintenance of nivolumab for the mRCC as per standard of care but added nab-paclitaxel in the hopes of obtaining a synergistic antitumor effect for the TNBC.

Historically, the mainstay of anticancer therapy was chemotherapy. Consequently, patients with synchronous tumors were treated with combination chemotherapy. With progress in research and drug development, the armamentarium of therapy has now been expanded to include multiple therapeutic agents such as chemotherapy, immunotherapy, genomic-directed targeted therapies, and hormonal therapies. While genomic sequencing of synchronous tumors could provide a single actionable mutation, these instances are uncommon. Individualized approaches, such as that employed in this case, in which a novel combination of immunotherapy and immunotherapy are used, may be critical for patient outcomes.
